# Association of D-dimer levels with vascular calcification in patients undergoing maintenance hemodialysis: a cross-sectional study

**DOI:** 10.3389/fmed.2026.1986142

**Published:** 2026-10-27

**Authors:** Zhijian Zhang, Xiran Zhang, Wei An, Weiwei Shan, Jing Mao, Jing Xue, Weiwei Li, Liang Wang

**Affiliations:** 1 Department of Nephrology, The Affiliated Wuxi People’s Hospital of Nanjing Medical University, Wuxi People’s Hospital, Wuxi Medical Center, Nanjing Medical University, Wuxi, China; 2 Department of Nephrology, The Second Affiliated Hospital of Soochow University, Suzhou, China

**Keywords:** cross-sectional study, D-dimer, maintenance hemodialysis, risk assessment, vascular calcification

## Abstract

**Background:**

Vascular calcification (VC) is common in patients undergoing maintenance hemodialysis (MHD) and is associated with adverse cardiovascular outcomes. D-dimer, a marker of coagulation and fibrinolysis, may reflect pathophysiological processes linked to VC, but its relationship with VC in MHD patients remains unclear.

**Methods:**

In this single-center cross-sectional study, 346 MHD patients were enrolled. VC was assessed by chest x—ray-based aortic arch calcification grading and defined as grade 0 vs. grades 1–3 for regression analyses. Multivariable logistic regression was used to evaluate the association between D-dimer and VC. A clinically applicable risk assessment model was developed. Internal validation used 1,000 bootstrap resamples in which the entire model-development process, including variable screening, was repeated, and the incremental value of D-dimer was evaluated by comparing models with and without D-dimer using optimism-corrected discrimination, calibration, overall performance, and decision curve analysis. Model performance was assessed by receiver operating characteristic analysis, calibration, and decision curve analysis.

**Results:**

D-dimer levels increased across VC grades (*P* < 0.001). After multivariable adjustment, D-dimer remained independently associated with VC; each 100 μg/L increase in D-dimer was associated with a 9.5% higher odds of VC (OR 1.095, 95% CI 1.014–1.182; *P* = 0.020). Age, hemoglobin, and calcium-based phosphate binder use were also independently associated with VC. The full model showed acceptable discrimination (apparent AUC 0.802, 95% CI 0.754–0.849; optimism-corrected AUC 0.770). Adding D-dimer to the multivariable model yielded only a modest, non-significant increase in AUC over the model without D-dimer (apparent AUC 0.802 vs. 0.787; optimism-corrected 0.770 vs. 0.756).

**Conclusions:**

In MHD patients, higher D-dimer levels were independently associated with VC. A model based on routinely available variables showed acceptable performance for individualized VC risk assessment, although the incremental discriminative value of D-dimer was modest.

## 1 Introduction

Vascular calcification (VC) is highly prevalent in patients undergoing maintenance hemodialysis (MHD) and represents an important contributor to cardiovascular morbidity and mortality in this population (1–3). Unlike passive mineral deposition, VC is now recognized as an active and tightly regulated process involving disordered mineral metabolism, chronic inflammation, oxidative stress, endothelial dysfunction, and osteogenic transformation of vascular smooth muscle cells (4, 5). Because cardiovascular disease remains the leading cause of death in patients with end-stage kidney disease, early identification of individuals at high risk of VC is of substantial clinical relevance.

Assessment of VC in clinical practice remains challenging. Computed tomography provides sensitive and quantitative evaluation of calcification burden, but its routine use is limited by cost, radiation exposure, and accessibility. By contrast, chest x-ray is inexpensive, widely available, and commonly performed in hemodialysis patients, making it a pragmatic tool for identifying radiographic vascular calcification in real-world settings (6, 7). Nevertheless, simple and accessible biomarkers that may help improve risk stratification are still needed.

D-dimer, a degradation product of cross-linked fibrin, is widely used as a marker of coagulation activation and secondary fibrinolysis. In patients receiving hemodialysis, D-dimer levels are frequently elevated, potentially reflecting a complex vascular milieu characterized by chronic inflammation, endothelial injury, oxidative stress, and a prothrombotic state (8–11). These pathophysiological processes overlap with mechanisms implicated in the development of VC, suggesting that D-dimer may be associated with vascular calcification in this population (12, 13). Although D-dimer has been extensively studied in thrombotic and cardiovascular disorders, its relationship with VC in MHD patients remains insufficiently defined.

Therefore, this study aimed to investigate the association between D-dimer levels and prevalent vascular calcification in patients undergoing maintenance hemodialysis and to develop a clinically applicable model for individualized risk assessment based on routinely available variables.

## 2 Methods

### 2.1 Study design and participants

This single-center cross-sectional study was conducted at Wuxi People's Hospital Affiliated to Nanjing Medical University between October 2023 and October 2024. Adult patients receiving maintenance hemodialysis were consecutively screened for eligibility. Patients were included if they were aged 18 years or older, had undergone maintenance hemodialysis for at least 3 months, and had complete clinical, laboratory, and imaging data. Patients were excluded if they had severe infection within the preceding month, active malignancy, cachexia, or missing key study data. No patient was pregnant at enrollment, and no female patient was receiving estrogen-containing therapy for contraception, hormone replacement, or osteoporosis. No patient had documented rheumatoid arthritis or antiphospholipid syndrome, and no episode of syncope, acute cerebrovascular event, or acute coronary event (acute myocardial infarction or unstable angina) occurred within the enrollment window. Silent (undocumented) infarction was not systematically screened; a small number of patients with clinically quiescent systemic lupus erythematosus or vasculitis were included. Only patients with complete data for the variables of interest were included in the final analysis; therefore, no imputation for missing data was performed. The study protocol was approved by the Ethics Committee of Wuxi People's Hospital Affiliated to Nanjing Medical University, and written informed consent was obtained from all participants.

### 2.2 Data collection

Demographic characteristics, clinical data, medication history, and laboratory parameters were extracted from the electronic medical record system. The variables collected included age, sex, smoking status, body mass index (BMI), systolic blood pressure (SBP), diastolic blood pressure (DBP), dialysis time, calcium-based phosphate binder use, hemoglobin, calcium, phosphorus, C-reactive protein (CRP), alkaline phosphatase (ALP), and serum creatinine (SCr). Cardiovascular disease was defined as a composite of physician-diagnosed ischemic heart disease, heart failure, or atrial fibrillation documented in the electronic medical record before enrollment. D-dimer was measured as a candidate predictor and recorded in μg/L. For all MHD patients, fasting venous blood samples were collected before the dialysis session after the long interdialytic interval. Blood samples were drawn from the vascular access before the dialysis session; no episodes of local phlebitis or access thrombosis were documented at the time of sampling. Laboratory testing was performed in the central laboratory of Wuxi People's Hospital according to standard clinical procedures. Clinical and laboratory variables were obtained from records closest to the date of chest x-ray assessment. All participants were ambulatory, community-dwelling outpatients attending regular maintenance hemodialysis sessions, and none were bedridden at enrollment. Physical activity level and sedentary time were not systematically quantified. Clinical and laboratory data were reviewed by trained investigators before analysis to ensure completeness and consistency.

### 2.3 Outcome definition and imaging assessment

VC was assessed using posteroanterior chest x-ray performed within 1 week of blood sampling. Chest x-ray was selected because it is routinely used during follow-up in MHD patients and offers the advantages of low cost, broad accessibility, and no additional imaging burden in routine clinical practice. Although computed tomography provides more precise quantification of calcification burden, chest x-ray-based aortic arch calcification grading has been widely used in dialysis populations as a pragmatic and clinically applicable method for risk stratification. Aortic arch calcification was graded according to the criteria described by Symeonidis (14) et al. as follows: grade 0, no visible calcification; grade 1, small punctate or a single thin calcified lesion; grade 2, one or more areas of thick calcification or circular calcification involving 50% or less of the aortic arch area; and grade 3, circular calcification involving more than 50% of the aortic arch area. Two grouping strategies were used. The four-grade classification (grades 0–3) was applied for baseline comparisons. For regression and risk assessment analyses, VC was defined as a binary outcome: no calcification (grade 0) vs. calcification (grades 1–3). All radiographic images were independently reviewed by two radiologists blinded to the clinical data, and any disagreement was resolved by consensus.

### 2.4 Statistical analysis

Statistical analyses were performed using R software (version 4.5.2). Continuous variables were tested for normality and are presented as mean ± standard deviation (SD) for normally distributed data or median (Q1, Q3) for non-normally distributed data, as appropriate, whereas categorical variables are presented as number (percentage). Group comparisons were performed using the independent-samples t test or one-way analysis of variance (ANOVA) for normally distributed continuous variables, the Mann–Whitney *U*-test or Kruskal–Wallis test for non-normally distributed continuous variables, and the chi-square test or Fisher's exact test for categorical variables, as appropriate. For the association analysis, five clinically relevant core variables (D-dimer, age, dialysis time, calcium, and phosphorus) were forced into the model regardless of their univariate significance to control for confounding of the D-dimer-VC association. The remaining candidate variables were screened by univariate logistic regression, and those with *P* < 0.10 were eligible for inclusion; the complete candidate set and screening results are provided in Supplementary Table S1. Multicollinearity among candidate predictors was assessed using the variance inflation factor (VIF), and no substantial multicollinearity was identified (all VIFs <5). D-dimer was rescaled by dividing by 100 before model fitting; therefore, the corresponding odds ratio represents the change in odds of VC for each 100 μg/L increase in D-dimer. A full multivariable logistic regression model was constructed for individualized risk assessment, and the nomogram, calibration curve, and decision curve analysis (DCA) were all derived from this same full model. Model discrimination was assessed using receiver operating characteristic (ROC) curves and quantified by the area under the curve (AUC) with 95% confidence intervals (CIs), and AUCs were compared using the DeLong test. Odds ratios and 95% CIs are Wald-type intervals derived from model coefficients and standard errors, with corresponding Wald *P* values. Additional performance metrics included the Brier score, calibration intercept, calibration slope, sensitivity, and specificity; sensitivity and specificity are reported at the Youden-derived threshold, which is presented as a descriptive operating characteristic rather than a clinically optimal decision threshold. Model calibration was evaluated using a calibration curve as well as calibration intercept and slope. Apparent and optimism-corrected performance estimates were distinguished. Internal validation used 1,000 bootstrap resamples in which the entire model-development process—including univariate screening and forcing of core variables—was repeated in every resample; optimism-corrected AUC, calibration intercept, calibration slope, and Brier score were obtained using Harrell's optimism correction, and the identical procedure, including the same screening rules, was applied to the model without D-dimer. The incremental value of D-dimer was assessed by the likelihood ratio test comparing models with and without D-dimer, and a formal likelihood ratio test for a D-dimer × age interaction was also performed. Decision curve analysis was performed for both the full model and the model without D-dimer across threshold probabilities of 0.05–0.95, with interpretation focused on the prespecified plausible threshold range of 0.10–0.90. The intended action was defined as initiating intensified cardiovascular risk management (including consideration of CT-based calcification scoring) for patients whose predicted probability exceeded the threshold, with treat-all and treat-none as reference strategies. As a sensitivity analysis for sparse data and model stability, Firth's penalized logistic regression was applied to the full model, and coefficient stability was assessed in 1,000 bootstrap resamples. To enhance model interpretability, SHAP was applied to the full model to quantify variable importance and visualize the contribution of individual predictors, particularly D-dimer, to both overall model output and individualized risk assessment. All tests were two-sided, and *P* < 0.05 was considered statistically significant.

## 3 Results

### 3.1 Baseline characteristics of the study population

The baseline characteristics of the 346 hemodialysis patients stratified by vascular calcification grade are shown in Table 1. Continuous variables were summarized as mean ± standard deviation for normally distributed data or median (Q1, Q3) for non-normally distributed data, as appropriate, whereas categorical variables were presented as number (percentage). Significant differences among the four groups were observed for age (*P* < 0.001), BMI (*P* = 0.035), DBP (*P* = 0.005), calcium-based phosphate binder use (*P* = 0.001), smoking status (*P* = 0.048), dialysis time (*P* < 0.001), and D-dimer levels (*P* < 0.001). Patients with more advanced vascular calcification tended to be older and had a longer dialysis time. In addition, BMI and DBP tended to be lower in patients with higher calcification grades, whereas calcium-based phosphate binder use was more frequent among those with more severe calcification. D-dimer levels differed significantly across vascular calcification grades, with higher median values observed in the calcification groups than in grade 0, suggesting a potential association between elevated D-dimer levels and vascular calcification. No significant differences were observed for sex, SBP, hypertension, diabetes, cardiovascular disease, drinking, blood urea nitrogen, SCr, ALP, albumin, calcium, phosphorus, parathyroid hormone, low-density lipoprotein, hemoglobin, CRP, or Kt/V (all *P* > 0.05).

**Table 1:** Baseline characteristics of hemodialysis patients stratified by vascular calcification grade.

Variable	Grade 0 (*n* = 105)	Grade 1 (*n* = 105)	Grade 2 (*n* = 72)	Grade 3 (*n* = 64)	*P* value
Age, years	46.00 (36.00, 57.00)	56.00 (46.00, 68.00)	60.50 (52.00, 75.00)	66.00 (49.50, 73.25)	<0.001
Male sex, *n* (%)	68 (64.76)	70 (66.67)	37 (51.39)	39 (60.94)	0.189
BMI, kg/m^2^	23.00 (20.40, 25.60)	22.70 (20.10, 24.90)	21.80 (19.73, 24.05)	21.50 (19.08, 23.72)	0.035
SBP, mmHg	144.65 ± 19.91	141.51 ± 21.58	144.29 ± 16.47	139.95 ± 20.79	0.387
DBP, mmHg	84.00 (75.00, 92.00)	78.00 (68.00, 83.00)	79.00 (71.50, 86.00)	77.50 (68.00, 87.00)	0.005
Hypertension, *n* (%)	85 (80.95)	83 (79.05)	53 (73.61)	47 (73.44)	0.554
Diabetes, *n* (%)	26 (24.76)	24 (22.86)	16 (22.22)	14 (21.88)	0.969
Calcium-based phosphate binder use, *n* (%)	6 (5.71)	14 (13.33)	8 (11.11)	17 (26.56)	0.001
Cardiovascular disease, *n* (%)	36 (34.29)	30 (28.57)	21 (29.17)	22 (34.38)	0.748
Smoking, *n* (%)	1 (0.95)	4 (3.81)	5 (6.94)	0 (0.00)	0.048
Drinking, *n* (%)	1 (0.95)	2 (1.90)	2 (2.78)	1 (1.56)	0.940
BUN, mmol/L	24.50 (18.90, 30.00)	24.20 (20.10, 28.30)	24.10 (18.00, 29.80)	24.80 (19.65, 29.27)	0.997
SCr, μmol/L	959.30 (751.80, 1,169.90)	984.70 (786.70, 1,142.80)	931.05 (712.22, 1,085.95)	879.60 (712.97, 1,031.23)	0.141
ALP, U/L	89.00 (72.00, 118.00)	93.00 (84.00, 112.00)	104.00 (78.50, 125.50)	108.00 (80.75, 133.75)	0.064
ALB, g/L	39.40 (37.20, 42.00)	38.90 (36.40, 41.90)	38.75 (36.40, 40.73)	38.55 (34.68, 40.52)	0.156
Dialysis time, months	54.00 (23.00, 97.00)	72.00 (27.00, 154.00)	79.50 (42.75, 129.25)	107.00 (56.00, 168.75)	<0.001
Calcium, mmol/L	2.17 (2.03, 2.31)	2.19 (2.07, 2.36)	2.17 (2.05, 2.31)	2.25 (2.04, 2.42)	0.188
Phosphorus, mmol/L	1.87 ± 0.54	1.77 ± 0.56	1.81 ± 0.65	1.96 ± 0.66	0.219
PTH, pg/mL	337.20 (177.90, 589.50)	284.30 (157.50, 527.40)	304.15 (144.82, 465.98)	318.10 (148.62, 564.08)	0.746
LDL, mmol/L	2.18 ± 0.82	2.21 ± 0.90	2.35 ± 0.74	2.26 ± 0.88	0.583
Hb, g/L	108.00 (94.00, 116.00)	113.00 (101.00, 124.00)	112.50 (97.75, 122.00)	109.00 (102.75, 121.00)	0.069
CRP, mg/L	1.50 (0.50, 6.10)	3.00 (0.50, 10.50)	2.90 (0.50, 11.15)	3.70 (0.50, 15.55)	0.124
D-dimer, μg/L	172.00 (108.00, 300.00)	266.00 (157.00, 492.00)	321.00 (155.50, 667.50)	322.00 (139.25, 768.75)	<0.001
Kt/V	1.49 (1.27, 1.73)	1.47 (1.32, 1.74)	1.56 (1.38, 1.76)	1.60 (1.39, 1.82)	0.179

Continuous variables are presented as mean ± standard deviation or median (Q1, Q3), as appropriate, and categorical variables are presented as *n* (%). Group comparisons were performed using one-way analysis of variance for normally distributed continuous variables, the Kruskal–Wallis test for non-normally distributed continuous variables, and the chi-square test or Fisher's exact test for categorical variables, as appropriate. Grade 0 indicates no vascular calcification; Grade 1, mild vascular calcification; Grade 2, moderate vascular calcification; and Grade 3, severe vascular calcification. BMI, body mass index; SBP, systolic blood pressure; DBP, diastolic blood pressure; BUN, blood urea nitrogen; SCr, serum creatinine; ALP, alkaline phosphatase; ALB, albumin; PTH, parathyroid hormone; LDL, low-density lipoprotein; Hb, hemoglobin; CRP, C-reactive protein; Kt/V, dialysis adequacy index.

### 3.2 Multivariable logistic regression analysis for vascular calcification

A full multivariable logistic regression model was constructed to identify factors associated with VC in hemodialysis patients (Table 2). Candidate variables were selected based on univariate analyses and clinical relevance, with D-dimer, age, dialysis time, calcium, and phosphorus forced into the model regardless of their univariate significance. After multivariable adjustment, D-dimer remained significantly associated with VC. Specifically, for each 100 μg/L increase in D-dimer, the odds of VC increased by 9.5% (odds ratio [OR], 1.095; 95% CI, 1.014–1.182; *P* = 0.020). Age was also independently associated with VC, with each additional year corresponding to a 5.6% increase in the odds of VC (OR, 1.056; 95% CI, 1.031–1.081; *P* < 0.001). In addition, hemoglobin (OR, 1.018; 95% CI, 1.001–1.035; *P* = 0.033) and calcium-based phosphate binder use (OR, 3.440; 95% CI, 1.261–9.383; *P* = 0.016) were positively associated with VC. CRP showed a marginal association with VC (OR, 1.018; 95% CI, 1.000–1.036; *P* = 0.050), whereas ALP showed a similar trend that did not reach statistical significance (OR, 1.005; 95% CI, 1.000–1.011; *P* = 0.054). By contrast, dialysis time, calcium, phosphorus, DBP, BMI, and SCr were not significantly associated with VC after multivariable adjustment (all *P* > 0.05). In a sensitivity analysis using Firth's penalized logistic regression, the calcium-based phosphate binder OR was 3.13 (95% CI, 1.19–8.22) vs. 3.44 unpenalized, and all other estimates were materially unchanged (Supplementary Table S2).

**Table 2:** Multivariable logistic regression analysis for factors associated with vascular calcification.

Variable	*β*	SE	OR	95% CI	*P* value
D-dimer (per 100 μg/L increase)	0.091	0.039	1.095	1.014–1.182	0.020
Age	0.054	0.012	1.056	1.031–1.081	<0.001
Dialysis time	0.002	0.002	1.002	0.997–1.006	0.394
Calcium	0.370	0.585	1.448	0.460–4.559	0.527
Phosphorus	0.137	0.246	1.147	0.709–1.857	0.576
DBP	−0.012	0.012	0.988	0.965–1.011	0.298
Hemoglobin	0.018	0.009	1.018	1.001–1.035	0.033
Calcium-based phosphate binder use	1.235	0.512	3.440	1.261–9.383	0.016
CRP	0.018	0.009	1.018	1.000–1.036	0.050
ALP	0.005	0.003	1.005	1.000–1.011	0.054
BMI	−0.055	0.036	0.946	0.882–1.015	0.120
SCr	0.001	0.001	1.001	1.000–1.002	0.159

Odds ratios (ORs) and 95% confidence intervals (CIs) were estimated using a full multivariable logistic regression model. CIs are Wald-type intervals based on model coefficients and standard errors (computed from unrounded estimates), and *P* values are Wald tests. Candidate variables were selected based on univariate analyses and clinical relevance, with D-dimer, age, dialysis time, calcium, and phosphorus forced into the model. D-dimer was rescaled by dividing by 100 before model fitting; therefore, the reported OR for D-dimer represents the change in odds of vascular calcification associated with each 100 μg/L increase in D-dimer. OR, odds ratio; CI, confidence interval; DBP, diastolic blood pressure; CRP, C-reactive protein; ALP, alkaline phosphatase; BMI, body mass index; SCr, serum creatinine.

### 3.3 Nomogram for individualized risk assessment of vascular calcification

Based on the full multivariable logistic regression model, a nomogram was developed to provide an individualized visual tool for estimating the probability of prevalent VC in hemodialysis patients (Figure 1). The nomogram incorporated D-dimer, age, dialysis time, calcium, phosphorus, DBP, hemoglobin, calcium-based phosphate binder use, CRP, ALP, BMI, and SCr, with D-dimer modeled per 100 μg/L increase. Each predictor was assigned a point value according to its relative contribution to the full model, and the total score was used to estimate the individual probability of VC. Among the variables included in the nomogram, age and D-dimer contributed prominently to the total point score, consistent with the multivariable logistic regression results. This graphical model enables clinicians to integrate multiple routinely available clinical and laboratory variables and generate individualized risk estimates for VC in hemodialysis patients.

**Figure 1:**
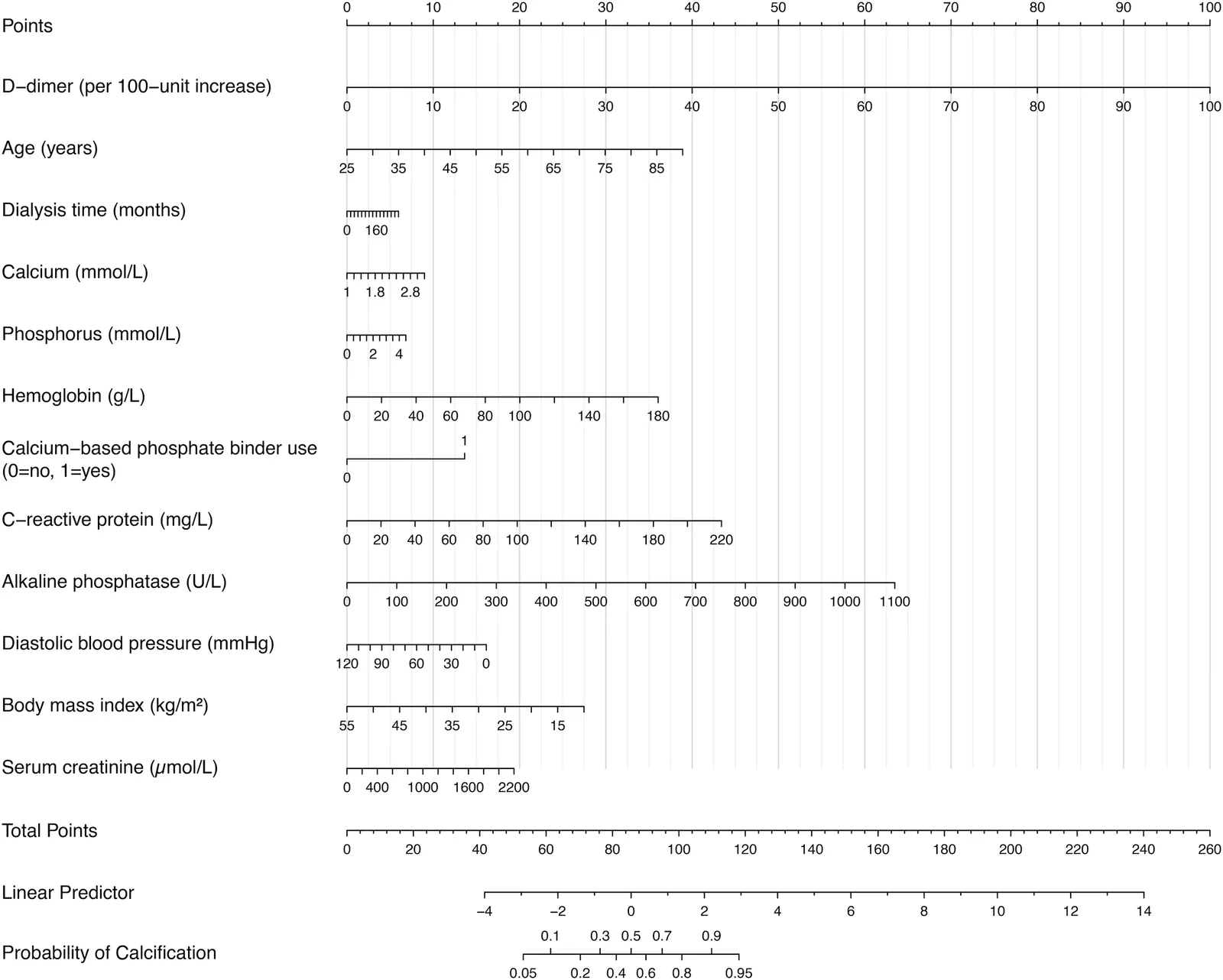
Nomogram for estimating the probability of prevalent vascular calcification in hemodialysis patients. A nomogram was constructed based on the multivariable logistic regression model to estimate the individual probability of prevalent vascular calcification. The nomogram included D-dimer, age, dialysis time, calcium, phosphorus, DBP, hemoglobin, calcium-based phosphate binder use, CRP, ALP, BMI, and SCr. D-dimer was modeled per 100 μg/L increase.

### 3.4 Discrimination, calibration, and clinical utility of the risk assessment models

The performance of the three models is shown in Figure 2. In ROC analysis, the full model demonstrated the highest discriminative ability for VC, with an AUC of 0.802 (95% CI, 0.754–0.849), compared with 0.649 (95% CI, 0.588–0.710) for the D-dimer-only model and 0.787 (95% CI, 0.736–0.837) for the model without D-dimer (Figure 2A). The D-dimer-only model showed significantly poorer discrimination than both the model without D-dimer (DeLong test, *P* = 3 × 10^−4^) and the full model (DeLong test, *P* < 0.001). Although the addition of D-dimer to the multivariable model was associated with a modest numerical increase in AUC compared with the model without D-dimer (0.802 vs. 0.787; *Δ*AUC = 0.015), this difference did not reach statistical significance by the DeLong test (Z = 1.786, *P* = 0.0741), whereas D-dimer significantly improved the model likelihood (likelihood ratio test, *P* = 0.002). The apparent Brier score of the full model was 0.162. After internal validation with 1,000 bootstrap resamples that repeated the entire variable-selection process, the optimism-corrected AUC was 0.770 (apparent 0.802), the corrected calibration intercept and slope were 0.09 and 0.84, and the corrected Brier score was 0.176. The model without D-dimer, validated with the identical procedure, yielded an optimism-corrected AUC of 0.756 (apparent 0.787), a corrected calibration slope of 0.85, and a corrected Brier score of 0.179 (Supplementary Table S3). In decision curve analysis, within the prespecified plausible threshold range of 0.10–0.90, the full model demonstrated higher net benefit than both the treat-all and treat-none strategies at every threshold (Figure 2C). Net benefit was closely similar between the full model and the model without D-dimer below about 0.35, marginally lower for the full model between 0.35 and 0.50, and higher for the full model above 0.51.

**Figure 2:**
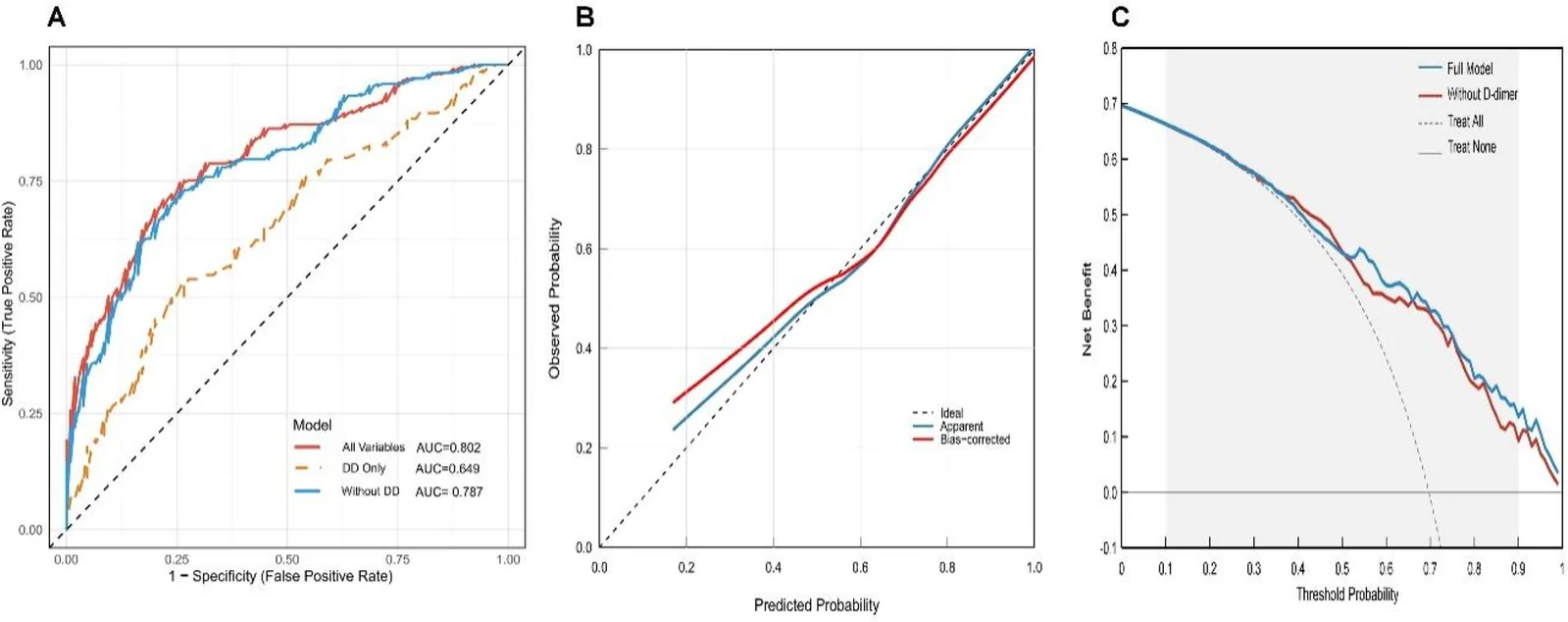
Discrimination, calibration, and decision curve analysis of the risk assessment models for vascular calcification. **(A)** Receiver operating characteristic (ROC) curves of the three models, including the full model (all variables), the D-dimer-only model, and the model without D-dimer. The AUC was 0.802 (95% CI, 0.754–0.849) for the full model, 0.649 (95% CI, 0.588–0.710) for the D-dimer-only model, and 0.787 (95% CI, 0.736–0.837) for the model without D-dimer. **(B)** Calibration curve of the nomogram derived from the full model, showing agreement between predicted and observed probabilities of vascular calcification, together with optimism-corrected performance estimates from 1,000 bootstrap resamples in which the entire model-development process was repeated (corrected AUC 0.770; corrected calibration intercept 0.09; corrected calibration slope 0.84; corrected Brier score 0.176). **(C)** Decision curve analysis comparing the full model and the model without D-dimer across threshold probabilities of 0.05–0.95. The intended action was initiating intensified cardiovascular risk management for patients exceeding the threshold probability; treat-all and treat-none are shown as reference strategies. Within the plausible threshold range of 0.10–0.90 (shaded), the full model showed higher net benefit than both reference strategies at every threshold. Relative to the model without D-dimer, net benefit was closely similar below about 0.35, marginally lower for the full model between about 0.35 and 0.50 (absolute difference up to 0.025), and higher for the full model above about 0.51 (absolute difference up to 0.057). The threshold of 0.7016 derived from the Youden index (sensitivity 0.7095, specificity 0.7810) is a descriptive operating characteristic rather than a clinically optimal decision threshold.

### 3.5 Model interpretability analysis based on SHAP

To improve the interpretability of the risk assessment model, SHAP analysis was performed for the same full multivariable model used to construct the nomogram (Figure 3). The SHAP summary plot showed the overall direction and magnitude of each predictor's contribution to model output (Figure 3A). Higher age and higher D-dimer levels were generally associated with positive SHAP values, indicating an increased estimated probability of VC. Dialysis time also made an important contribution to model output. The feature importance plot ranked predictors according to their mean absolute SHAP values (Figure 3B). Age was the most influential variable, followed by D-dimer and dialysis time. Other variables, including BMI, SCr, ALP, DBP, hemoglobin, calcium, phosphorus, CRP, and calcium-based phosphate binder use, showed relatively smaller contributions. These findings further support the prominent role of age and D-dimer in model output and enhance the interpretability of the full model for potential clinical application.

**Figure 3:**
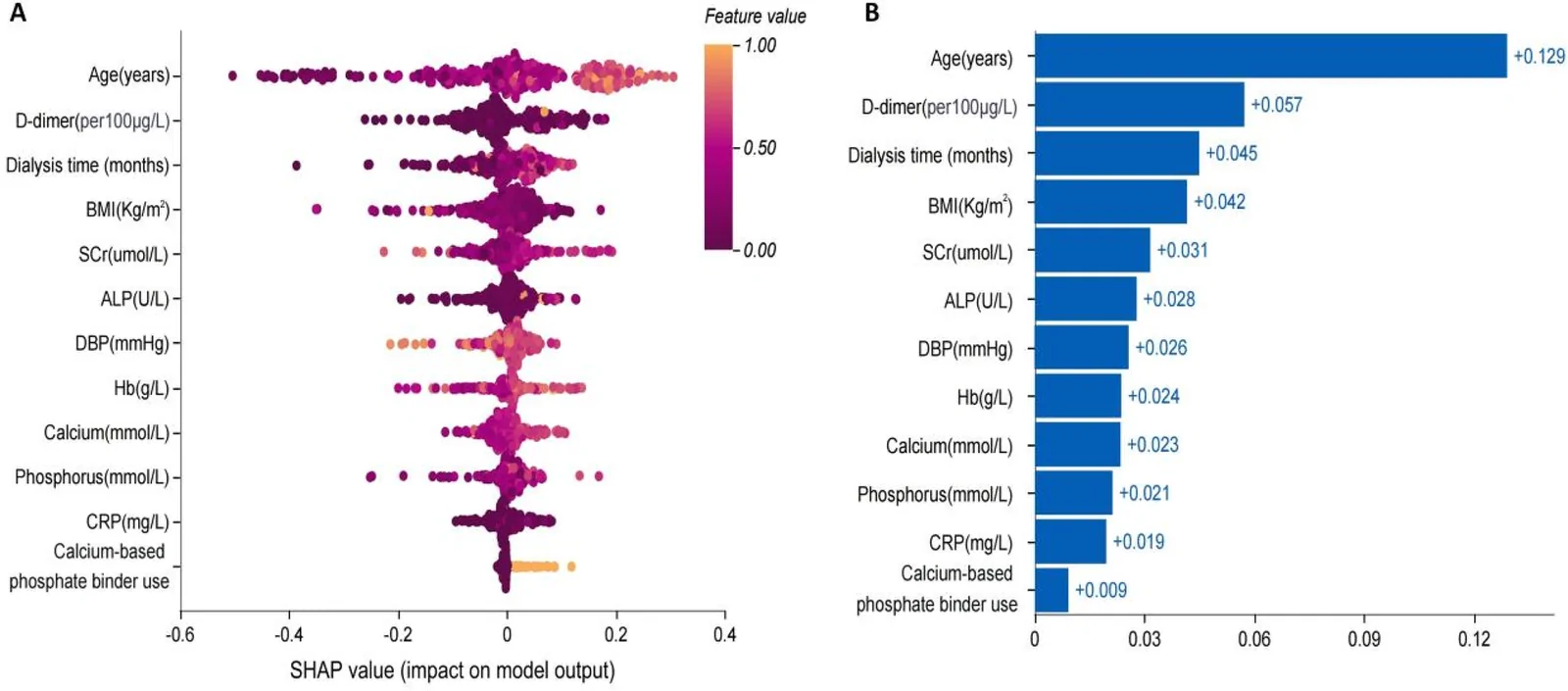
SHAP summary plot and feature importance of the risk assessment model for vascular calcification. **(A)** SHAP summary plot showing the distribution of SHAP values for each predictor included in the model. Each dot represents one patient. The *x*-axis indicates the SHAP value, reflecting the impact of each variable on model output, with positive values indicating a higher estimated probability of vascular calcification and negative values indicating a lower estimated probability. Color represents the relative feature value from low to high. **(B)** Bar plot of mean absolute SHAP values showing the relative importance of each predictor in the model. Age, D-dimer, and dialysis time were the top three contributors to model output.

### 3.6 Individualized explanation of a high-risk patient

To further explore whether the association between D-dimer and VC varied by age, a formal likelihood ratio test for a D-dimer × age interaction was performed in the full model; the interaction was not significant (*P* = 0.571), indicating that the association did not differ significantly across age. To illustrate the model output at the individual level, a SHAP waterfall plot was generated for a representative high-risk patient (Figure 4). The model assigned a high estimated probability of VC to this patient, with age, D-dimer, ALP, phosphorus, SCr, and dialysis time contributing positively to the model output, whereas BMI showed a negative contribution. These findings provide an intuitive explanation of how multiple clinical variables jointly influenced the individualized risk estimate and further support the relevance of D-dimer in personalized VC risk assessment.

**Figure 4:**
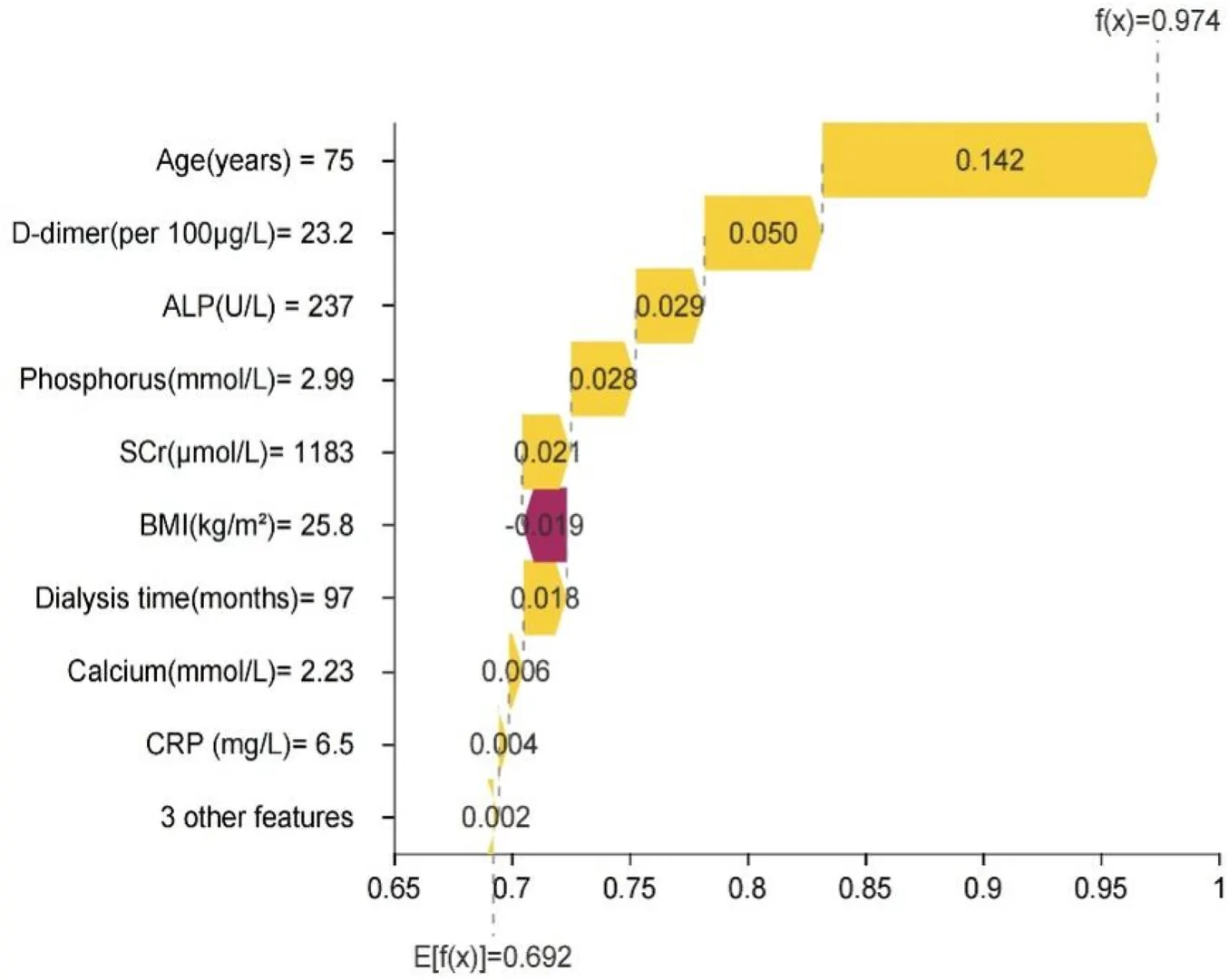
SHAP waterfall plot for a representative high-risk patient. SHAP waterfall plot showing the contribution of each predictor to the final model output. D-dimer is shown as per 100 μg/L (rescaled by dividing by 100 before model fitting). Yellow bars indicate increased estimated risk; dark red bars indicate decreased estimated risk.

## 4 Discussion

In this single-center cross-sectional study of patients undergoing maintenance hemodialysis, higher D-dimer levels were independently associated with a greater likelihood of prevalent vascular calcification after adjustment for demographic, clinical, and laboratory factors. Age, hemoglobin, and calcium-based phosphate binder use were also independently associated with vascular calcification. Based on these routinely available variables, we developed a nomogram for individualized risk assessment, and the full model demonstrated acceptable overall performance.

The association between D-dimer and vascular calcification is biologically plausible. In the hemodialysis population, D-dimer levels are frequently elevated as a result of impaired renal clearance, chronic inflammation, and repeated vascular injury, reflecting an ongoing prothrombotic state (15). This prothrombotic state is both a consequence and a driver of endothelial injury and inflammation, which in turn promote osteogenic differentiation of vascular smooth muscle cells, extracellular matrix remodeling, and progressive mineral deposition within the vascular wall (16–20). From this perspective, elevated D-dimer may serve not merely as a marker of coagulation activity but as an integrated indicator of the adverse vascular pathophysiology that predisposes to calcification. Elevated D-dimer has also been associated with subclinical atherosclerosis and arterial stiffness in some studies of populations with preserved kidney function (21), although this association has not been consistently observed (22); evidence specific to patients with vascular calcification and preserved kidney function remains limited. Whether the association observed in our MHD cohort extends to non-uremic populations therefore warrants further study in a separate cohort with preserved kidney function.

Our findings also highlight the contribution of conventional clinical factors to vascular calcification in hemodialysis patients. Age remained one of the strongest contributors in the multivariable model, consistent with cumulative vascular injury and progressive arterial degeneration over time. Calcium-based phosphate binder use was positively associated with vascular calcification, which is clinically plausible given concerns that excess calcium exposure may accelerate ectopic calcification in susceptible individuals (23, 24). Hemoglobin was also positively associated with vascular calcification. Although this finding is less straightforward, it may reflect complex interactions involving treatment intensity, erythropoiesis-stimulating agent use, iron status, nutritional condition, and residual confounding (25, 26). Thus, the positive association between hemoglobin and VC likely reflects the influence of treatment-related or nutritional factors rather than a direct pro-calcific effect of hemoglobin itself. By contrast, dialysis time, calcium, phosphorus, DBP, BMI, and SCr were not independently associated with vascular calcification after multivariable adjustment, suggesting that their effects may be partly mediated by, or shared with, other correlated clinical factors. With respect to body size, although D-dimer levels are generally higher in obesity, BMI was lower in patients with more advanced calcification and showed an inverse, non-significant association with VC after adjustment; this reversed pattern may reflect the malnutrition–inflammation complex and the reverse-epidemiology phenomenon well described in dialysis populations (27). Regarding blood pressure, lower diastolic pressure in patients with more advanced calcification is consistent with wide pulse pressure and increased large-artery stiffness—a hemodynamic state that accompanies arterial calcification rather than plausibly causing it—and DBP was not independently associated with VC after multivariable adjustment. In addition, because D-dimer levels rise with age, we examined the age-adjusted association: D-dimer remained significantly associated with VC after adjustment for age (OR per 100 μg/L, 1.073; 95% CI, 1.002–1.148; *P* = 0.044).

A notable finding was that although D-dimer remained independently associated with vascular calcification, its addition to the multivariable model produced only a modest increase in discrimination, which was not statistically significant by the DeLong test. This is not unexpected. A variable may retain independent statistical significance in regression analysis while offering limited incremental value in global discrimination, particularly when the base model already includes strong predictors such as age and other established clinical variables. Accordingly, the clinical utility of D-dimer in this setting may lie less in materially improving AUC and more in complementing routine clinical assessment and supporting individualized risk stratification. This perspective is consistent with the view that D-dimer, as a marker of coagulation activation, may reflect underlying vascular pathology even when its additive predictive value over conventional risk factors is limited (28). Beyond discrimination, D-dimer significantly improved the model likelihood, and within decision curve analysis the full model provided higher net benefit than the model without D-dimer across the plausible threshold range, suggesting that the clinical contribution of D-dimer lies mainly in risk stratification rather than in global discrimination. In other words, D-dimer may still be clinically informative even if its incremental predictive contribution beyond conventional variables is modest.

Vascular calcification was assessed using chest x-ray-based aortic arch calcification grading rather than CT. While this pragmatic approach is suitable for clinical risk stratification, it predominantly detects overt macroscopic calcification and is less sensitive to early or diffuse disease. Accordingly, our findings reflect radiographically evident vascular calcification rather than the full systemic burden measurable by CT, a distinction that should be considered when contextualizing our results against studies using more sensitive imaging modalities. From a clinical perspective, the proposed model is intended as a screening and stratification aid rather than a diagnostic test. It could be applied at the time of routine chest x-ray evaluation—already part of standard follow-up in many dialysis units—without additional testing or cost, to estimate an individual patient's probability of prevalent aortic arch calcification. A high predicted probability would prompt actions that are feasible within current care pathways, including intensification of cardiovascular risk management (blood pressure and volume control, optimization of mineral metabolism, and attention to inflammation), consideration of transthoracic echocardiography, and prioritization for CT-based calcification scoring when resources permit; a low predicted probability would support continuing routine follow-up. Importantly, whether use of the model changes downstream clinical decisions or improves outcomes has not been established by this cross-sectional study and requires prospective evaluation. The integration of SHAP-based interpretation into the nomogram further enhances its transparency, allowing clinicians to visualize which factors drive an individual patient's risk estimate and facilitating shared decision-making at the bedside.

Several limitations should be considered. First, the cross-sectional design precludes inference regarding causality or temporal sequence. The observed association between D-dimer and vascular calcification should therefore not be interpreted as evidence of prediction for future progression or of a direct mechanistic role. Second, vascular calcification was assessed by chest x-ray rather than computed tomography, which may have reduced sensitivity for milder or earlier disease and may not fully capture overall calcification burden. Third, although internal validation suggested acceptable model stability, external validation was not performed, and the generalizability of the nomogram to other hemodialysis populations remains uncertain. Fourth, despite adjustment for multiple clinically relevant covariates and the absence of substantial multicollinearity, residual confounding cannot be excluded. Unmeasured factors such as vitamin D analog use, calcimimetic therapy, anticoagulant treatment, dialysis prescription, residual kidney function, autoimmune activity (a small number of patients with clinically quiescent SLE or vasculitis were included) and other comorbidities may have influenced both D-dimer levels and vascular calcification risk. Sedentary behavior and physical activity level were not systematically quantified in this study and may represent a further source of residual confounding, as sedentary behavior can elevate D-dimer levels. Catheter use has been associated with higher D-dimer levels independent of anticoagulation, and vascular access type was not included in the multivariable model; this may represent a source of residual confounding. Calcium-based phosphate binder use was sparse among patients without calcification, and this estimate therefore carries uncertainty. Finally, the incremental discrimination provided by D-dimer beyond conventional variables was limited and should be interpreted cautiously.

## 5 Conclusion

Higher D-dimer levels were independently associated with prevalent vascular calcification in patients undergoing maintenance hemodialysis. A multivariable model incorporating D-dimer and routinely available clinical variables showed acceptable performance for individualized risk assessment. However, the additional discriminative value of D-dimer beyond conventional predictors was modest. Further multicenter prospective studies are needed to validate these findings and to clarify the role of D-dimer in vascular calcification risk stratification in the hemodialysis population.

## Data Availability

The raw data supporting the conclusions of this article will be made available by the authors, without undue reservation.
