# Supplementary material for: Association of D-dimer levels with vascular calcification in patients undergoing maintenance hemodialysis: a cross-sectional study
Source: Front Med (Lausanne). 2026 Oct 27;13:1986142. doi: 10.3389/fmed.2026.1986142 (PMC13747767; doi:10.3389/fmed.2026.1986142)
Supplement: Supplementary file 1 [file Datasheet1.pdf]

SUPPLEMENTARY MATERIAL

Association of D-Dimer Levels With Vascular Calcification in Patients Undergoing Maintenance Hemodialysis: A Cross-Sectional Study

Zhijian Zhang[1†], Xiran Zhang[1†], Wei An[1†], Weiwei Shan[1], Jing Mao[1], Jing Xue[1], Weiwei Li[2*], Liang Wang[1*]

[1]Department of Nephrology, The Affiliated Wuxi People's Hospital of Nanjing Medical University, Wuxi People's Hospital, Wuxi Medical Center, Nanjing Medical University, Wuxi, Jiangsu 214023, P.R. China.

[2]Department of Nephrology, The Second Affiliated Hospital of Soochow University, Suzhou, Jiangsu 215004, China.

[*]Correspondence: Liang Wang (wangliang_wuxi@126.com); Weiwei Li (weiweisdfey@163.com). [†]These authors contributed equally to this work.

Supplementary Tables

Table S1. Univariate screening of the complete candidate set (n = 346).
Table S2. Firth penalized sensitivity analysis of the full multivariable model.
Table S3. Internal validation with 1,000 bootstrap resamples repeating the entire variable-selection process.

Supplementary Table S1. Univariate screening of the complete candidate set (n = 346).

Variable	OR	95% CI	P value	Eligible (P < 0.10)
Age (per year)	1.065	1.045–1.086	<0.001	Yes
DBP (per mmHg)	0.969	0.951–0.987	<0.001	Yes
Dialysis time (per month)	1.007	1.003–1.011	<0.001	Yes
D-dimer (per 100 μg/L)	1.125	1.043–1.215	0.002	Yes
Hemoglobin (per g/L)	1.017	1.005–1.030	0.007	Yes
Calcium-based phosphate binder use	3.186	1.305–7.777	0.011	Yes
CRP (per mg/L)	1.018	1.002–1.035	0.030	Yes
ALP (per U/L)	1.006	1.001–1.011	0.030	Yes
BMI (per kg/m²)	0.941	0.888–0.996	0.036	Yes
SCr (per μmol/L)	0.999	0.999–1.000	0.077	Yes
Albumin (per g/L)	0.959	0.911–1.009	0.106	No
Calcium (per mmol/L)	1.974	0.765–5.095	0.160	No
Smoking	4.034	0.505–32.258	0.188	No
Kt/V	1.445	0.831–2.510	0.192	No
SBP (per mmHg)	0.993	0.982–1.005	0.244	No
Hypertension	0.742	0.420–1.312	0.305	No
PTH (per pg/mL)	1.000	0.999–1.000	0.344	No
LDL-C (per mmol/L)	1.122	0.852–1.479	0.412	No
Cardiovascular disease	0.833	0.511–1.356	0.462	No
Drinking	2.203	0.254–19.094	0.473	No
Phosphorus (per mmol/L)	0.900	0.614–1.319	0.589	No
Diabetes	0.877	0.513–1.501	0.633	No
BUN (per mmol/L)	0.997	0.968–1.028	0.864	No

Note. OR, odds ratio; CI, confidence interval; ALP, alkaline phosphatase; BMI, body mass index; BUN, blood urea nitrogen; CRP, C-reactive protein; DBP, diastolic blood pressure; Kt/V, single-pool Kt/V (dialysis dose); LDL-C, low-density lipoprotein cholesterol; PTH, parathyroid hormone; SBP, systolic blood pressure; SCr, serum creatinine.

Estimates are from univariable logistic regression with Wald-type inference; ORs are expressed per unit increase as indicated in parentheses. Vascular calcification was defined as aortic arch calcification grade 1–3 versus grade 0 on chest radiography (105 patients with grade 0 and 241 with grade 1–3). Five clinically relevant core variables (D-dimer, age, dialysis time, calcium, and phosphorus) were forced into the multivariable model regardless of univariate significance; of these, D-dimer, age, and dialysis time also met the $P < 0.10$ threshold. Variables with $P < 0.10$ were eligible for inclusion, and the final multivariable model retained 12 variables: the 5 core variables plus the 7 eligible candidates DBP, hemoglobin, calcium-based phosphate binder use, CRP, ALP, BMI, and SCr.

Supplementary Table S2. Firth penalized sensitivity analysis of the full multivariable model (n = 346).

Variable	Unpenalized OR (95% CI)	Firth-penalized OR (95% CI)
D-dimer (per 100 μg/L)	1.095 (1.014–1.182)	1.082 (1.008–1.160)
Age (per year)	1.056 (1.031–1.081)	1.053 (1.029–1.078)
Dialysis time (per month)	1.002 (0.997–1.006)	1.002 (0.997–1.006)
Calcium (per mmol/L)	1.448 (0.460–4.559)	1.375 (0.445–4.247)
Phosphorus (per mmol/L)	1.147 (0.709–1.857)	1.129 (0.704–1.809)
DBP (per mmHg)	0.988 (0.965–1.011)	0.989 (0.967–1.011)
Hemoglobin (per g/L)	1.018 (1.001–1.035)	1.018 (1.001–1.034)
Calcium-based phosphate binder use	3.440 (1.261–9.383)	3.127 (1.189–8.220)
CRP (per mg/L)	1.018 (1.000–1.036)	1.016 (0.999–1.033)
ALP (per U/L)	1.005 (1.000–1.011)	1.004 (0.999–1.009)
BMI (per kg/m²)	0.946 (0.882–1.015)	0.951 (0.889–1.018)
SCr (per μmol/L)	1.001 (1.000–1.002)	1.001 (1.000–1.002)

Note. OR, odds ratio; CI, confidence interval; ALP, alkaline phosphatase; BMI, body mass index; CRP, C-reactive protein; DBP, diastolic blood pressure; SCr, serum creatinine.

All estimates are from the same multivariable logistic model (241 patients with vascular calcification among 346 patients) and are expressed per unit increase as indicated in parentheses. Unpenalized estimates are maximum-likelihood estimates; Firth-penalized estimates are from Firth's penalized (ridge-type) logistic regression, which reduces small-sample bias and is reported as a sensitivity analysis. Confidence intervals are Wald-type intervals computed on the respective unpenalized or penalized log-likelihood.

Supplementary Table S3. Internal validation of the full model and of the model without D-dimer (1,000 bootstrap resamples; Harrell optimism correction).

Metric	Full model			Without D-dimer	
	Apparent	Optimism	Corrected	Apparent	Corrected
AUC	0.802	+0.031	0.770	0.787	0.756
Calibration slope	1.000	+0.157	0.843	1.000	0.852
Calibration intercept (CITL)	0.000	-0.085	0.085	0.000	0.088
Brier score	0.162	-0.014	0.176	0.166	0.179

Note. AUC, area under the receiver operating characteristic curve; CITL, calibration-in-the-large; SD, standard deviation.

The full model contained 12 predictors; the comparison model was identical except that D-dimer was omitted (11 predictors). The identical validation procedure was applied to both models: 1,000 bootstrap resamples of the whole dataset in which the entire model-development process, including variable selection, was repeated, with Harrell's optimism correction. Apparent values are estimated in the development sample; corrected values equal the apparent value minus the estimated optimism. The calibration intercept is the calibration-in-the-large and the calibration slope describes the agreement between predicted and observed probabilities (a value of 1 indicates perfect calibration). The Brier score is a measure of overall accuracy in which lower values indicate better performance.
